# Biomarkers for Clinical and Incipient Tuberculosis: Performance in a TB-Endemic Country

**DOI:** 10.1371/journal.pone.0002071

**Published:** 2008-04-30

**Authors:** Ajay Wanchu, Yuxin Dong, Sunil Sethi, V. P. Myneedu, Arthur Nadas, Zhentong Liu, John Belisle, Suman Laal

**Affiliations:** 1 Department of Internal Medicine, Post Graduate Institute of Medical Education and Research, Chandigarh, India; 2 Department of Medical Microbiology, Post Graduate Institute of Medical Education and Research, Chandigarh, India; 3 Department of Microbiology, Lala Ram Sarup Institute of Tuberculosis and Respiratory Diseases, New Delhi, India; 4 Department of Microbiology, Immunology and Pathology, Colorado State University, Fort Collins, Colorado, United States of America; 5 Department of Pathology, New York University School of Medicine, New York, New York, United States of America; 6 Department of Microbiology, New York University School of Medicine, New York, New York, United States of America; 7 Institute of Environmental Medicine, New York University School of Medicine, New York, New York, United States of America; 8 Veterans Affairs Medical Center, New York, New York, United States of America; Oregon Health & Science University, United States of America

## Abstract

**Background:**

Simple biomarkers are required to identify TB in both HIV^−^TB^+^ and HIV^+^TB^+^ patients. Earlier studies have identified the *M. tuberculosis* Malate Synthase (MS) and MPT51 as immunodominant antigens in TB patients. One goal of these investigations was to evaluate the sensitivity and specificity of anti-MS and –MPT51 antibodies as biomarkers for TB in HIV^−^TB^+^ and HIV^+^TB^+^ patients from a TB-endemic setting. Earlier studies also demonstrated the presence of these biomarkers during incipient subclinical TB. If these biomarkers correlate with incipient TB, their prevalence should be higher in asymptomatic HIV^+^ subjects who are at a high-risk for TB. The second goal was to compare the prevalence of these biomarkers in asymptomatic, CD4^+^ T cell-matched HIV^+^TB^−^ subjects from India who are at high-risk for TB with similar subjects from US who are at low-risk for TB.

**Methods and Results:**

Anti-MS and -MPT51 antibodies were assessed in sera from 480 subjects including PPD^+^ or PPD^−^ healthy subjects, healthy community members, and HIV^−^TB^+^ and HIV^+^TB^+^ patients from India. Results demonstrate high sensitivity (∼80%) of detection of smear-positive HIV^−^TB^+^ and HIV^+^TB^+^ patients, and high specificity (>97%) with PPD^+^ subjects and endemic controls. While ∼45% of the asymptomatic HIV^+^TB^−^ patients at high-risk for TB tested biomarker-positive, >97% of the HIV^+^TB^−^ subjects at low risk for TB tested negative. Although the current studies are hampered by lack of knowledge of the outcome, these results provide strong support for the potential of these biomarkers to detect incipient, subclinical TB in HIV^+^ subjects.

**Conclusions:**

These biomarkers provide high sensitivity and specificity for TB diagnosis in a TB endemic setting. Their performance is not compromised by concurrent HIV infection, site of TB and absence of pulmonary manifestations in HIV^+^TB^+^ patients. Results also demonstrate the potential of these biomarkers for identifying incipient subclinical TB in HIV^+^TB^−^ subjects at high-risk for TB.

## Introduction

Over 90% of the ∼8.8×10^6^ tuberculosis (TB) cases that occur annually live in resource-constrained countries where TB is endemic and the diagnosis is based on microscopic examination of smears prepared directly from the patient specimens (mostly sputum) for acid-fast bacilli (AFB) [1], [2]. While microscopy identifies the highly infectious multibacillary patients, its diagnostic performance varies depending on the diligence and the work-load of the microscopist, it requires multiple specimens (and patient visits) which leads to significant drop-out of infectious patients, and takes several days to provide results under programmatic conditions [1]. As the HIV-epidemic has taken root in the TB-endemic countries, the inadequacies of microscopy-based TB diagnosis have been exacerbated since the immunosuppression of cellular responses in the dually-infected patients results in diminished cavity formation, and consequently, greater proportion of both smear-negative TB and extrapulmonary TB (EPTB) [3]. Biomarkers for TB that can be adapted to robust, point-of-care and affordable user-friendly formats that can replace the AFB smear-based diagnosis and rapidly identify both HIV^−^TB^+^ and HIV^+^TB^+^ patients are urgently required [3].

Efforts to exploit antibodies as biomarkers for diagnosis for TB were unsuccessful for decades [4] but promising antigens have been identified in recent years [5], [6], [7], [8], [9], [10], [11], [12], [13], [14]. Our labs have used screening of immunoblots of 2-D fractionated *Mycobacterium tuberculosis* (*M. tb)* culture-filtrate proteins [8], [9], [15], microarrays of *M. tb* cytosolic and culture-filtrate proteins [16] and *M. tb* DNA expression libraries [17], [18] with sera from TB patients and *M. tb*-infected animals at different stages of TB to identify several immunodominant antigens. While some of the immunodominant antigens (eg, 38kDa PhoS protein) are specific to cavitary TB [6], [8], [19], others, such as the 81-kDa Malate Synthase (MS) (Rv1837c) and the 29-kDa MPT51 (Rv3803c) elicit antibodies in patients at different stages of clinical TB (smear-negative or positive TB; non-cavitary or cavitary TB) and in different classes of TB patients (HIV^−^TB^+^ or HIV^+^TB^+^) [8], [9], [20], [21]. Moreover, retrospective stored sera that were obtained from HIV^+^TB^+^ patients during the months prior to clinical manifestation of TB when they were asymptomatic (subclinical TB) were demonstrated to contain antibodies to these antigens [9]. Since these antibodies were not detected in sera from PPD^−^, PPD^+^ or HIV^+^TB^−^ subjects, these results suggested that the emergence of anti-MS and/or MPT51 antibodies could serve as biomarkers for incipient TB in asymptomatic HIV^+^ subjects [6], [9].

The currently available commercial antibody biomarkers fail to provide acceptable sensitivity and specificity in TB-endemic settings [4]. It is important that the new candidate biomarkers be evaluated in TB-endemic countries before further development is considered. The first goal of the current studies was to evaluate the sensitivity and specificity of anti-MS and –MPT51 antibodies as biomarkers for TB in HIV^−^TB^+^ and HIV^+^TB^+^ patients from a TB-endemic setting. This has been done with subjects from India which has an exceptionally high TB burden (250–300/100,000) [22] and where 50–80% of the population is estimated to be latently infected with *M. tb* [23], [24].

As in other TB-endemic countries, 60–70% of the HIV^+^ subjects in India develop TB as their AIDS-defining opportunistic infection. Since earlier studies with retrospective sera from HIV^+^TB^+^ patients from the US indicated that anti-MS and/or MPT51 antibodies may serve as biomarkers for incipient subclinical TB, this suggested that a higher proportion of HIV^+^ subjects at high-risk for TB would test positive for these biomarkers compared to HIV^+^ subjects at low-risk for TB. The second goal of the current studies was to test this hypothesis by comparing the prevalence of these biomarkers in asymptomatic, CD4^+^ T cell-matched HIV^+^TB^−^ subjects at low-risk or high-risk for TB.

## Materials and Methods

### Study populations

Data reported in these studies are compiled from results obtained with serum specimens tested over several years. Approval for the studies was obtained from the Lala Ram Sarup Institute for Tuberculosis and Respiratory Diseases (LRSI) ethics committee and subsequently from the Indian Council for Medical Research (ICMR) in 2001. Patients recruited from LRSI after obtaining approvals gave written consent. Approval for the studies was also obtained from the ethics committee at Post Graduate Institute for Medical Education and Research (PGIMER), and subsequently from the ICMR in 2004. Aliquots of sera obtained from 40 HIV^−^TB^+^ patients and all HIV^+^TB^+^ and HIV^+^ subjects with oral consent (1998–2001) and from 36 HIV^−^TB^+^ patients and 38 healthy subjects with written consent (2004) for other investigations were tested for the current studies after ICMR approvals were obtained. No information that links individuals with serum specimens is available. The sera from the HIV^+^ patients and healthy subjects at the Veterans Affairs Medical Center (VAMC) were obtained under consent forms approved by the VAMC and/or New York University School of Medicine (NYUSoM) IRB committees.

To evaluate the sensitivity and specificity of anti-MS and/or MPT51 antibodies as biomarkers for TB in HIV^−^TB^+^ and HIV^+^TB^+^ patients in a TB-endemic setting, sera from the following groups of individuals were included in these studies.

#### a) PPD^+^ and PPD^−^ healthy subjects

(n = 52) 35 HIV^−^PPD^+^ and 17 HIV^−^PPD^−^ individuals were included; 24 of 35 PPD^+^ individuals were immigrants from TB-endemic countries (India, China, Cameroon) working in the VAMC; 8 were bled within 2–12 weeks of arrival. Most of these individuals were also BCG vaccinated. Eleven PPD^+^ subjects, none of who was BCG vaccinated were from US or Europe. Seven PPD^−^ individuals were immigrants and 10 were US-born.

#### b) Healthy community controls

(HCC; n = 38) Physicians, scientists, nurses, technicians from PGIMER, with no risk for HIV were enrolled as healthy community controls.

#### c). Non-HIV TB patients

(HIV^−^TB^+^; n = 160): 138 TB patients were AFB sputum smear-positive; 124 of these were enrolled from LSRI. Fourteen sputum smear-positive and 22 smear-negative patients were from PGIMER. AFB cultures are not routinely performed in PGIMER but the 22 smear-negative TB patients were *M. tb* culture confirmed for the purposes of this study.

#### d). HIV-infected TB patients

(HIV^+^TB^+^; n = 61). Of the 60 smear-positive HIV^+^TB^+^ patients enrolled at PGIMER, 50 presented with TB and were bled prior to initiation of anti-retroviral therapy (ART) or anti-TB therapy (ATT). The remaining 10 HIV^+^TB^+^ patients (who were not on ART) developed TB during follow-up in the HIV clinic. Thirty two of the 60 (53%) patients had normal chest X-rays, 12 (20%) showed infiltration, 4 each (6.5%) had cavitary lesions or military TB, 3 (5%) showed signs of interstitial infiltration with PCP, 3 (5%) had pleural effusions and 2 (2%) showed presence of nodular lesions. Thirty four (57%) patients had EPTB (mostly lymph-node TB); of these 31 had normal chest X-rays. The CD4^+^ T cells in these patients ranged from 18–548/ul.

Eleven HIV^+^TB^−^ patients progressed to HIV^+^TB^+^ during follow-up. For 10 of these patients, 13 specimens obtained prior to manifestation of TB (and 10 drawn at time of TB diagnosis) were available. For the eleventh HIV^+^TB^+^ patient, 3 sera obtained prior to diagnosis of TB were available but no serum specimen was obtained at TB diagnosis. The sera obtained prior to manifestation and diagnosis of TB is referred to as SCTB sera.

To compare the prevalence of anti-MS and/or MPT51 antibodies in subjects who are at high-risk for TB with those at low risk for TB, sera were obtained from the following groups of subjects:

#### e) HIV-infected subjects at high-risk for TB

(HIV^+^TB^−^, HR; n = 96). These were untreated HIV^+^ patients from PGIMER whose chest X-rays showed no abnormalities and had no clinical symptoms suggestive of TB. Their CD4+ T cells ranged from 11–1090/ul. PPD skin test or Quantiferon testing was not done in these subjects but earlier studies have demonstrated a high incidence (50–80%) of latent infection in the Indian population.

#### f). HIV-infected subjects at low-risk for TB

(HIV^+^TB^−^, LR; n = 73). These were HIV^+^ asymptomatic individuals on ART with no history or clinical suspicion of TB recruited from the VAMC, NY. Their CD4^+^ T cells ranged from 108–862/ul.

### Antigens

Cloning and expression of the genes encoding MS and MPT51 and purification of the recombinant proteins was as previously described [9].

### ELISA

Recombinant purified MS or MPT51 were coated at 4 ug/ml (50 ul/well) and the sera tested at 1∶50 dilution for MS or 1∶25 dilution for MPT51 [9]. A mixture of alkaline phosphatase-conjugated Protein A (1∶2000, Sigma, St. Louis, MO) and anti-IgA (1∶1000, Sigma) was used to detect the antigen-bound antibodies. The Invitrogen Amplification System (Invitrogen, Carlsbad, CA) was used for color development in a majority of the experiments but during a time period when it was not available, PhosphoGLO AP substrate (KPL, Gaithersburg, MD) was used. With the latter substrate, after it's addition to the ELISA-plate wells, the contents of the wells were transferred to black plates (Microfluor 2 Black, Thermo Milford, MA) and the plates read within 15 minutes in a Lumimark Plus luminometer (BioRad, Hercules, CA). Comparative studies with a defined set of serum specimens confirmed that the 2 substrates yielded same results (data not shown). The mean optical density (490 nm) or the mean relative light units (RLU) obtained with the PPD^+^/PPD^−^ healthy individuals + 3 standard deviations (SD) was used as cut-off to determine positive reactivity. Each specimen was tested 2–4 times; specimens which were consistently positive, positive 2/3 or 3/4 times were considered positive. This serum bank was collected over several years and the sera were tested in batches as they were obtained (always with ∼same numbers of control sera); delta-OD or delta RLU (OD/RLU of specimen - cut-off in that assay) was computed from different ELISA assays to normalize results. The ELISA assays were done in the US laboratory in the context of the same control sera.

### Statistical Analysis

Mann-Whitney test and the t-test were used to compare the reactivity of sera from PPD^−^ or PPD^+^ healthy subjects with the two antigens. The Pearson sample correlation was calculated to determine the correlation between presence or absence of antibodies and CD4^+^T cell numbers in HIV^+^TB^+^ patients.

## Results

### Anti-MS and anti-MPT51 antibodies in PPD^+^ and PPD^−^ Healthy Control subjects

**Figure 1:**
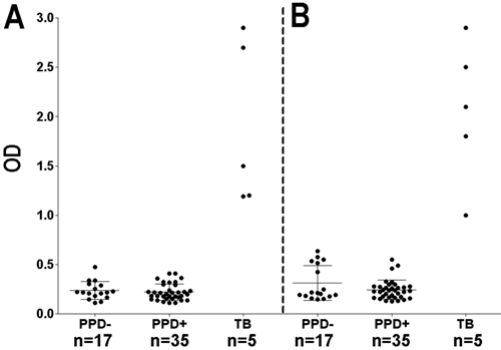
Reactivity of sera with MS and MPT51: Sera from HIV^−^PPD^−^ and HIV^−^PPD^+^ healthy subjects and TB patients were tested for reactivity with MS (A) and MPT51 (B). Mean OD (optical density)±standard deviation is depicted.

The reactivity of the sera from 17 PPD^−^ healthy controls and the 35 PPD^+^ healthy controls with MS and MPT51 was compared by ELISA (Fig 1). Sera from 5 AFB sputum smear-positive TB patients known to be positive for reactivity with the 2 antigens was used as positive controls for the ELISA [9]. When the OD values for the 2 groups of healthy subjects were compared by Mann-Whitney test (or the t-test) there was no significant difference between them (p-value 0.5955 for MS and 0.5712 for MPT51). Thus healthy subjects who may be latently infected with *M. tb* and/or were BCG vaccinated lacked antibodies to the 2 antigens.

### Anti-MS and anti-MPT51 antibodies in TB patients and healthy controls

**Figure 2:**
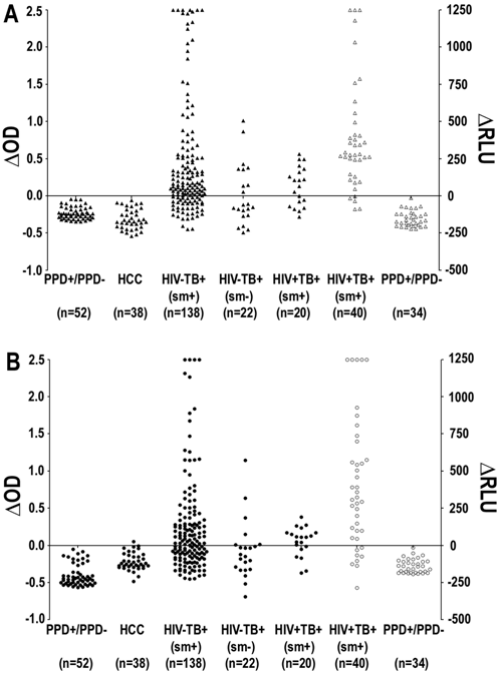
Presence of anti-MS or anti-MPT51 antibodies in TB patients: Sera from healthy PPD+/PPD^−^ subjects, healthy community controls (HCC) and TB patients was tested for presence of anti-MS (A) or anti-MPT51 (B) antibodies. Delta OD (optical density) represents the OD obtained with the individual serum specimen minus the mean OD plus 3 standard deviations obtained with the PPD^+^/PPD^−^ healthy subjects. Filled symbols represent results from ELISA assays in which the Invitrogen substrate was used. Delta RLU (relative light units) represents relative light units obtained with the individual serum specimen minus the mean RLU plus 3 standard deviations obtained with sera from PPD^+^/PPD^−^ healthy subjects. Hollow symbols indicate results from ELISA assays in which the PhosphoGLO AP substrate was used. Numbers in parenthesis indicate number of subjects in each group.

**Figure 3:**
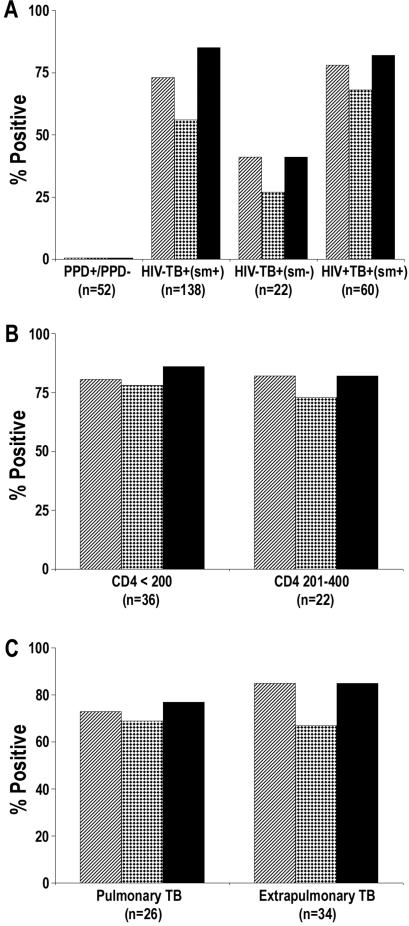
Anti-MS and/or anti-MPT51 antibodies as biomarkers for TB in different categories of patients: Presence of the two biomarkers in smear-positive HIV^−^TB^+^, smear-negative HIV^−^TB^+^ and smear-positive HIV^+^TB^+^ patients (A). Presence of the biomarkers in HIV^+^TB^+^ patients with CD4^+^ T cell numbers <200/ul or between 201–400 (B). Presence of the biomarkers in HIV^+^TB^+^ patients with pulmonary TB or extrapulmonary TB (C). In all figures, bars with diagonal lines represent anti-MS antibodies, bars with dots represent anti-MPT51 antibodies and additive reactivity with both biomarkers is represented by solid black bars. Numbers in parenthesis indicate number of subjects in each group.

The mean OD of all 52 PPD^−^/PPD^+^ subjects plus 3 SD was used as cut-off to determine positive responses in TB patients. Based on this cut-off no HCC showed the presence of anti-MS antibodies and 1/38 showed presence of anti-MPT51 antibodies (specificity >97%). In contrast, anti-MS and anti-MPT51 antibodies were detected in 75% (103/138) and 59% (82/138) of the smear-positive HIV^−^TB^+^ patients respectively (Fig 2A and 2B). Together, these biomarkers identified 80% (111/138) of the smear-positive patients (Fig. 3A). Forty-four percent (9 of 22) smear-negative TB patients also tested positive with either one or both biomarkers (Fig 2A, 2B and 3A). Among the 60 smear-positive HIV^+^TB^+^ patients, 47 (78%) had anti-MS and 41 (68%) anti-MPT51 antibodies (Fig 2A and 2B); 82% (49/60) of the patients exhibited presence of either one or both biomarkers in their sera (Fig 3A).

The results from HIV^+^TB^+^ patients were analyzed based on their CD4^+^ T cell status. Of the 36 patients with CD4^+^ T cells <200/ul, 29 (81%) had anti-MS and 28 (77%) had anti-MPT51 antibodies; 31 of 36 (86%) patients tested positive for at-least one of the 2 biomarkers (Fig 3B). Of the 22 HIV^+^TB^+^ patients with CD4^+^ T cell numbers between 201–400/ul, 18 (82%) possessed anti-MS and/or MPT51 antibodies (Fig 3B). Two patients with CD4^+^ T cells >400/ul were excluded from this analysis. There was no statistical correlation between the presence or absence of antibodies and the CD4^+^ T cell numbers when the Pearson sample correlation was calculated (r = −0.060).

Of 34 HIV^+^TB^+^ patients with EPTB, 29 had anti-MS and 23 had anti-MPT51 Abs (Fig 3C); either one or both biomarkers were demonstrated in 85% (29/34) patients. Nineteen patients with PTB had anti-MS and 18 anti-MPT51 antibodies; 20/26 (77%) tested positive for either one or both biomarkers (Fig 3C). A vast majority of the patients with normal chest X-rays (31/34) had EPTB. Either one or both biomarkers were detected in 28 of 32 (87%) HIV^+^TB^+^ patients with normal chest X-rays and in 26/28 (89%) of patients with any radiological abnormality (data not shown).

**Figure 4:**
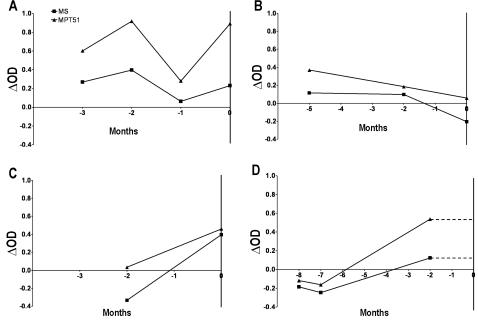
Presence of anti-MS and anti-MPT51 antibodies during subclinical TB: Presence of the two biomarkers in retrospective sera obtained during the months prior to manifestation of clinical TB in 4 HIV^+^TB^+^ patients is shown. Time 0 represents the time point at which clinical TB was diagnosed; negative values refer to months preceding time 0. Delta OD (optical density) represents the OD obtained with the individual serum specimen minus the mean OD plus 3 standard deviations obtained with the PPD^+^/PPD^−^ healthy subjects in the ELISA. For patient depicted in ‘D’, no specimen was obtained at time of clinical diagnosis of TB.

The retrospective specimens obtained 2–48 months prior to clinical TB from the 11 HIV^+^TB^+^ patients were tested for these biomarkers. For 8 of 11 patients, the retrospective specimens were obtained within 6 months prior to TB diagnosis and retrospective sera from 7/8 patients were antibody-positive (Table 1, Fig 4). For 3 patients the retrospective specimens were obtained 6–48 months prior to TB, and these specimens tested negative (Table 1).

**Table 1:** Presence of anti-MS &/or –MPT51 antibodies during SCTB in HIV^+^TB^+^ patients

PATIENT	AT –TB	SCTB	TIME BETWEEN SCTB SPECIMEN & TB (MONTHS)
1	Positive	Positive	2
2	Serum NA	Positive	3
3	Positive	Positive	3
4	Positive	Positive	3
5	Positive	Positive	4
6	Positive	Positive	4
7	Positive	Positive	5
8	Positive	Negative	6
9	Positive	Negative	7
10	Positive	Negative	13
11	Positive	Negative	48

NA: Not available

Together these results demonstrate that anti-MS and/or MPT51 antibodies are present in sera from ∼80% of the smear-positive HIV^−^TB^+^ and HIV^+^TB^+^ patients but absent in sera from the vast majority of PPD^+^ subjects and endemic controls. The sensitivity of the biomarkers is unaffected by the CD4^+^ T cell loss that accompanies progressive HIV-infection and is similar in patients with PTB or EPTB. Finally, these biomarkers are present in retrospective sera that were obtained from HIV^+^ TB^+^ patients during 2–6 months prior to the clinical manifestation of TB.

### Anti-MS and/or MPT51 antibodies in asymptomatic HIV^+^TB^−^ subjects at high-risk or low-risk for TB

**Figure 5:**
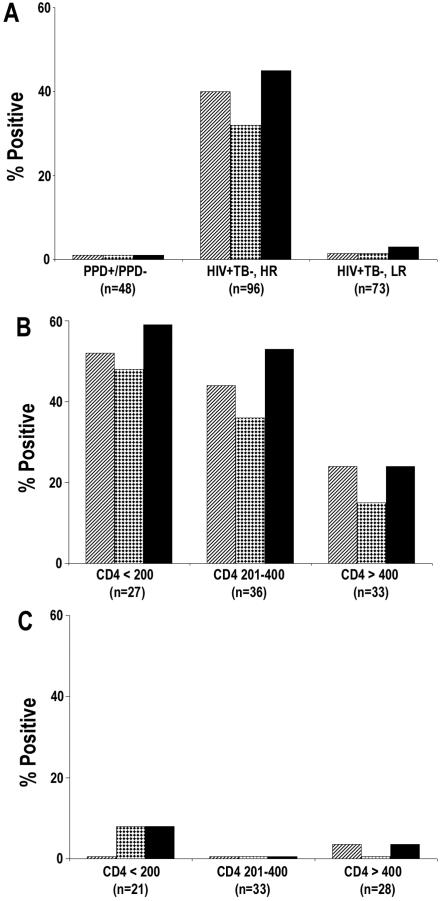
Presence of anti-MS and/or anti-MPT51 antibodies in HIV^+^TB^−^ subjects at high-risk or low-risk for TB: Sera from healthy PPD^+^/PPD^−^ subjects, asymptomatic HIV^+^TB^−^ subjects at high-risk for TB (HR) and asymptomatic HIV^+^TB^−^ subjects at low-risk (LR) for TB was tested for presence of anti-MS and -MPT51 antibodies (A). Presence of these biomarkers in sera from HIV^+^TB^−^, HR subjects with different CD4^+^ T cell numbers (B), and in sera from HIV^+^TB^−^, LR subjects with different CD4^+^ T cell numbers (C). In all figures, bars with diagonal lines represent anti-MS and bars with dots represent anti-MPT51 antibodies. Additive reactivity of both biomarkers is represented by solid black bars. Numbers in parenthesis indicate number of subjects in each group.

The presence of these biomarkers was tested in asymptomatic HIV^+^TB^−^ subjects from India who are at a high-risk for active infection with *M. tb* (HIV^+^TB^−^, HR) and HIV^+^TB^−^ subjects from the US who are a low risk for TB (HIV^+^TB^−^, LR). Sera from 40% (38 of 96) of the HIV^+^TB^−^, HR subjects exhibited presence of anti-MS and 32% (31 of 96) anti-MPT51 antibodies; either one or both biomarkers were demonstrated in sera from 45% (43/96) of these subjects (Fig 5A). In contrast, 97% (63/65) of the HIV^+^TB^−^, LR subjects were negative for both biomarkers (Fig 5A).

There was an inverse correlation between the CD4^+^ T cells and presence of these biomarkers in sera from HIV^+^TB^−^, HR subjects. Of the 96 HIV^+^TB^−^, HR subjects, 27 (28%) had CD4^+^ cells <200/ul and 16 (59%) were biomarker positive (Fig 5B); 36 subjects (38%) had CD4^+^ T cells ranging from 201–400/ul and 19 (52%) were biomarker positive (Fig 5B), 33 (34%) subjects had CD4^+^ T cells >400/ul and 8 (24%) were positive (Fig 5B). Thus, the proportion of biomarker-positive subjects increased with decreasing CD4^+^ T cell numbers. There was no correlation between these biomarkers and CD4^+^ T cells in the HIV^+^TB^−^, LR subjects (Fig 5C).

Thus, while anti-MS and/or MPT51 antibodies are rarely detected in sera from asymptomatic HIV^+^ subjects who are at a low-risk for TB; these biomarkers are detected in sera from a significant proportion of HIV^+^ asymptomatic subjects who are at the high risk for TB.

## Discussion

These results demonstrate that anti-MS and/or -MPT51 antibodies are sensitive and specific biomarkers that identify ∼80% of both HIV^−^TB^+^ and HIV^+^TB^+^ AFB smear positive patients from a TB-endemic country. Importantly, the sensitivity of these biomarkers is unaffected by factors that compromise the performance of the currently used diagnostic tools in HIV^+^ patients, i.e. reduced cavity formation, extrapulmonary location and immunosuppression of cellular immune responses [25]. A similar sensitivity of anti-MS antibodies was observed in HIV^+^TB^+^ patients from the US [9], [21], and has been demonstrated in other cohorts [11], [12], [13]. The high sensitivity provided by MPT51 in HIV^+^TB^+^ patients has also been reported [26]. Thus, although studies with some *M. tb* antigens suggested that antibody responses are poor or absent in HIV^+^TB^+^ patients [27], [28], [29], our earlier studies provided evidence for a defined subset of *M. tb* antigens (including MS and MPT51) eliciting antibodies in these patients, and several independent labs have now confirmed the potential of anti-MS and-MPT51 antibodies as sensitive biomarkers for TB diagnosis in these patients [9], [11], [12], [13], [21], [26], [30]. This is important since the performance of biomarkers requiring intact cellular responses (DTH or γ-interferon) is likely to be compromised by the dysregulated cellular immunity in the HIV^+^ patients [31], [32].

These biomarkers were absent in sera from >97% of the endemic controls and in sera from >97% of the HIV^+^ subjects who are at a low-risk for TB. The high specificity in PPD^+^ subjects, HIV^+^TB^−^ patients, and patients with lung cancer, pneumonia and non-TB lung infections has also been reported by other investigators [11], [12], [26]. The absence of these biomarkers in sera from PPD^+^ subjects, and in healthy subjects from India and from other TB endemic countries confirms the correlation of their presence with active infection with *M. tb*. MS is an enzyme of the glyoxalate pathway required for intracellular growth and replication of *M. tb* [33]. Also, the *M. tb* MS has adapted to become a secreted and surface-localized laminin-binding adhesin that enhances the adherance of *M. tb* to pulmonary epithelial cells [34]. These roles in intracellular survival and in pathogenesis provide an explanation for the expression of MS (and therefore presence of anti-MS antibodies) when active bacterial replication is ongoing *in vivo*. MPT51 is also an adhesin of *M. tb,* likely expressed during active replication of the bacteria, as suggested by its presence in culture filtrates of *M. tb* [8]. The absence of these biomarkers in sera from healthy PPD^+^ subjects and from BCG-vaccinated subjects indicates that immunostimulatory levels of the 2 proteins are achieved *in vivo* only during active bacterial replication.

Although a sensitivity of ∼80% is not sufficient to replace microscopy, no other biomarkers that provide this high sensitivity in smear positive HIV^−^TB^+^ as well as HIV^+^TB^+^ patients, and can identify both pulmonary and extrapulmonary TB even when the CD4^+^ T cells have been significantly diminished in the latter subjects have been reported [10], [30]. MS and MPT51 are 2 of the 12 immunodominant *M. tb* antigens that elicit antibodies in both HIV^−^TB^+^ and HIV^+^TB^+^ patients [8] and it is likely that inclusion of one or two additional antigens from this subset will further enhance the sensitivity to levels (90–95%) that are required of any test that can replace microscopy [35]. Several additional novel immunodominant antigens that were identified in our studies are currently under investigation [16], [17].

Anti-MS and/or MPT51 antibodies were also detected in sera from ∼45% of the HIV^−^TB^+^ AFB smear-negative patients, none of who would be identified by microscopy. These results are similar to those reported earlier with smear-negative HIV^−^TB^+^ patients from the US [21]. Thus, at the current performance level, in a setting where 40% of the patients present with smear-negative TB, these biomarkers would identify 66% of all TB cases, compared to 60% being recognized by the smear test alone. In this regard, although no smear-negative HIV^+^TB^+^ patients were tested in the current studies; we have earlier demonstrated the presence of these biomarkers in sera from ∼80% of the smear-negative HIV^+^TB^+^ patients [9], [21]. The ability to detect patients with smear-negative TB, and especially the high sensitivity in smear-negative HIV^+^TB^+^ patients is important since concurrent HIV-infection reduces pulmonary cavitation and sputum bacillary load while accelerating the progression of TB [3], [22], [25], [36]. The higher sensitivity of these biomarkers in smear-negative HIV^+^TB^+^ patients is likely related both to a greater *in vivo* bacterial burden as well as the generalized activation of B cell responses, including the antigen-specific B cells, caused by HIV-infection [37], [38], [39].

As was reported with retrospective sera from US HIV^+^TB^+^ patients [6], [9], these biomarkers were also demonstrated in the retrospective sera obtained 2–6 months prior to TB diagnosis from HIV^+^TB^+^ patients from India. Given the slow generation time of *M. tb*, the *in vivo* replication of bacteria would be expected to be initiated several weeks/months prior to the bacterial burden reaching levels that are detectable by conventional means. The ability of the immune system to detect the *in vivo* replication provides an important tool for detection of active infection while it is sub-clinical. Interestingly, the biomarkers were detected in sera that were obtained ∼6 months (or less) prior to clinical TB; the period of incipient TB has been estimated to be 2–9 months in HIV^+^ subjects in other studies [40].

Approximately 75–80% of the millions of HIV-infected patients in sub-Saharan Africa and Southeast Asia are co-infected with *M. tb*, and as a result 50–70% of the HIV^+^ subjects in these countries develop TB [22], [40], [41]. This is in contrast to the US where even before the availability of ART, only 3–4% of the HIV^+^ patients developed TB. Recent studies from other TB endemic countries (Tanzania, South Africa, and Peru) have reported that passive case finding fails to recognize a significant proportion of HIV^+^TB^+^ patients 40, 42, 43. Based on the high sensitivity of anti-MS and –MPT51 Abs as biomarkers for TB in paucibacillary HIV^+^TB^+^ patients [9], [21], and their presence in retrospective sera from HIV^+^TB^+^ patients [9], we hypothesized that a higher proportion of HIV^+^TB^−^ subjects at high-risk for TB will test positive for these biomarkers as compared to HIV^+^TB^−^ subjects at low-risk for TB. When CD4^+^ T cell-matched asymptomatic HIV^+^TB^−^ HR subjects from India and HIV^+^TB^−^ LR subjects from the US were compared, ∼45% of the former and <5% of the latter tested biomarker positive. The risk for TB increases as the T-cell immunity wanes, and as expected, the proportion of biomarker-positive HIV^+^TB^−^ HR subjects increased with decreasing CD4^+^ T cells. Although these cross-sectional studies are hampered by the lack of knowledge of the final outcome, these results suggest the presence of significant unrecognized TB in the HIV^+^ subjects from this endemic setting. It is likely that a proportion of the biomarker-positive HIV^+^TB^−^, HR subjects would have been identified had cultures been performed while others had truly incipient TB. Interestingly, in a recent pilot study from Gugulethu township in Cape town in which nebulized induced sputum specimens obtained from 140 consecutively enrolled HIV^+^ patients with advanced immunodeficiency were tested using fluorescence microscopy and automated liquid culture (MGIT-960,Becton Dickinson, USA), 30% of the patients yielded positive *M. tb* cultures (Dr. Stephen D. Lawn: personal communication). Prospective studies to evaluate the predictive potential of these biomarkers for TB, and for discriminating between truly latent and incipient subclinical TB in HIV^+^ subjects are planned. The accurate identification of paucibacillary as well as incipient TB will impact clinical decisions on treatment (INH prophylaxis versus multidrug therapy) and time of ART initiation. Also, early treatment of active TB may slow progression of HIV-infection.

The results of the current studies demonstrate the potential of anti-MS and –MPT51 as biomarkers both for diagnosis of TB and for detection of incipient *M. tb* infection in HIV^+^ patients in a TB-endemic setting. Although the sensitivity of diagnosis needs to be enhanced, and the final panel of selected antigens will have to be adapted to rapid formats, the current studies provide encouraging results for feasibility of the development of a rapid, simple and accurate test for TB diagnosis.
